# A quantum-like cognitive approach to modeling human biased selection behavior

**DOI:** 10.1038/s41598-022-13757-2

**Published:** 2022-12-29

**Authors:** Aghdas Meghdadi, M. R. Akbarzadeh-T, Kurosh Javidan

**Affiliations:** 1 Department of Electrical Engineering, Center of Excellence on Soft Computing and Intelligent Information Processing, Ferdowsi University of Mashhad, Mashhad, Iran; 2 Department of Physics, Ferdowsi University of Mashhad, Mashhad, Iran

**Keywords:** Computer science, Quantum physics

## Abstract

Cognitive biases of the human mind significantly influence the human decision-making process. However, they are often neglected in modeling selection behaviors and hence deemed irrational. Here, we introduce a cognitive quantum-like approach for modeling human biases by simulating society as a quantum system and using a Quantum-like Bayesian network (QBN) structure. More specifically, we take inspiration from the electric field to improve our recent entangled QBN approach to model the initial bias due to unequal probabilities in parent nodes. Entangled QBN structure is particularly suitable for modeling bias behavior due to changing the state of systems with each observation and considering every decision-maker an integral part of society rather than an isolated agent. Hence, biases caused by emotions between agents or past personal experiences are also modeled by the social entanglement concept motivated by entanglement in quantum physics. In this regard, we propose a bias potential function and a new quantum-like entanglement witness in Hilbert space to introduce a biased variant of the entangled QBN (BEQBN) model based on quantum probability. The predictive BEQBN is evaluated on two well-known empirical tasks. Results indicate the superiority of the BEQBN by achieving the first rank compared to classical BN and six QBN approaches and presenting more realistic predictions of human behaviors.

## Introduction

With the advent of the Internet of Things and social networks, the reformation of the digital stock market, intelligent navigation and traffic systems, disaster management, and energy consumption, in which humans are central interlayers, comes a critical need to develop better human–machine interfaces and reach more realistic models of human selection behavior. What presents a significant challenge here is the bias behavior observed in these human-centered systems that are nonlinear, uncertain, complex, and disobey the 'rational' decision-making processes dictated by the traditional probabilistic structures^1,2^. Despite extensive studies on the mathematical modeling of decision-making in different fields^3^, modeling the bias behavior in the decision-making process remains an open problem.

Past studies have led to different decision-making models, including Bayesian networks (BN)^4^, prospect theory^5^, Markov decision^6^, expected utility (EU)^7^, game theory^8^, Dempster-Shafer theory (DS)^9^, and fuzzy decision making^10^. Although these models and their variants have achieved significant performance, more psychological studies indicate behaviors that cannot be explained by the mentioned classical methods^2,11^. Recently some irrational observations, such as order effect^12^, conjunction/disjunction error^13^, or violation of classical probability rules, including the sure-thing principle (STP)^14^ and the total probability law (TPL)^15^, revealed the need for revising the mathematical structure used in classical models^12,16^. For this purpose, different quantum-like decision theories are proposed based on quantum probability structure in the Hilbert space^16^. Using the term quantum-like in these models means that these theories do not deal with quantum modeling of the decision-making in the brain nor about using quantum computers. They use only the structure of QP to develop the mathematical structure of modeling in the human mind. The main idea of this study is to propose a biased variant of quantum-like decision-making approaches to provide more accurate predictions of human behavior and to justify a broad range of decision-making paradoxes.

Recently, Quantum Probability (QP) has been applied extensively in various fields such as engineering^17^, economics^18^, psychology^19^, biology^20^, and decision-making^21^, referred to as the Second Quantum Revolution^22^. Applying quantum probability in decision-making theories was introduced by Aerts et al.^23^. They show that classical probability (CP) cannot successfully model the role of the observer in the social sciences. Over the years, many studies have been done to develop this idea. Busemeyer et al.^19^ model the dynamical evolution of the decision-making process in Hilbert space based on solving the Schrödinger equation. Khrennikov et al.^24^ use probability interference, the key concept in quantum mechanics (QM), to explain the violation of the STP. They use contextual probabilities and wave functions in modeling decision-making processes inspired by the double-slit experiment. Then, Wang et al.^25^ propose a quantum question order model, known as the QQ model, to describe the order effect in decision-making. They introduce the q-test value to recognize the order effect in empirical experiments and prove the Quantum Question (QQ) equality under some circumstances to predict the selection of humans in different order of questions. This model is developed by Ozawa et al.^26^ to correct for the order effect in the data and determine the “genuine” distribution of the opinions in the poll.

Another quantum-like decision approach is a quantum-like Bayesian network (QBN) that can predict human selection behavior as well as justify the contradiction of CP's rules^14^. In this approach, Moreira et al. replace QP with the CP in the BN structure and propose a heuristic function for estimating the interference effect. Recently, we introduced a predictive entangled QBN model (PEQBN)^27^ motivated by quantum information theory (QIT). According to QIT, a composite system is entangled when unknown relationships link the individual components as a single entity. We simulate a social system as a composite quantum one and model the effect of society on each decision-maker (DM) by simulating BN’s nodes as entangled wave functions. In this model, we introduce a social entanglement concept inspired by quantum entanglement. The effect of society on the decision-making process, using a quantum-like approach, is also studied by Tsarev^28^.

Recently, a new perspective of quantum mechanics has been proposed in physics and philosophy, which discusses the ontological and epistemological architecture of quantum mechanics^29^ and presents the subjective probability interpretation of wave functions^30^. These theories such as QBism^30^, introduced by Fuchs, consider that many, but not all, aspects of quantum formalism are subjective in nature. According to the Fuchs view, the wave functions are mathematical abstractions that help us to organize our thinking like the calculus of probabilities, instead of being considered as real entities like ripples on a pond^31^. Recently, some quantum-like theories of cognition have been proposed along with these perspectives^32,33^. The development of mathematical structures of such theories can lead to a better understanding of the concept of the wave function and thus the development of quantum-like decision models in the future.

On the other hand, there are some cognitive and neuroscience studies on bias behavior in the human mind. The term *cognitive bias* was first introduced in the decision-making domain by Tversky and Kahneman^2^. Afterward, various types of cognitive bias behaviors such as anchoring, availability heuristic, representativeness heuristic, and many more^1^ have been confirmed during experimental studies. Also, there are some neurological studies on bias behavior due to past experiences or emotions in decision making. Sacré et al.^34^ confirm that rationality in financial decision-making may compete with a bias that reflects past outcomes. In another neurological study, Nielson et al.^35^ study irrational economic decisions. Kesteren et al.^36^ verify that a preceding judgment biases the current one if the preceding and current items are of the same perceptual category. Also, the impact of moods and emotions experienced on choice behavior and social decision-making is studied by Engelmann et al.^37^ and Farolfi et al.^38^ based on behavioral and neural research. Ravi et al.^39^ study the accumulating evidence that prior knowledge about expectations plays an important role in perception. Their findings demonstrate substantial deviations from the ideal Bayesian detector, which could be a sign that the brain utilizes a heuristic approximation of the Bayesian inference. They discuss the power of Bayesian-like heuristics in the brain, as well as their limitations.

Quantum-like Bayesian network structure is particularly suitable for modeling bias behaviors because this structure can model the role of observer/measurements on the state of DM’s mind using the phase parameters in the complex Hilbert space. It is noted that there are only a few neurological studies on Bayesian inference^40^ in the literature. Matsumori et al. ^41^ introduce a bias Bayesian inference and discuss its neural implementation. Asano et al.^42^ consider irrational updating in classical Bayesian inference and define quantum-like bias operations due to some psychological element acting on the mental state. Recently, Wojciechowski et al.^43^ applied quantum probability to justify constructive biases in clinical judgment.

Here, we extend the PEQBN model and introduce a biased entangled quantum-like Bayesian network (BEQBN) as a predictive decision-making approach inspired by the electric fields, the entanglement concept, and cognitive studies. We consider different types of biased behaviors due to the emotion between agents such as friendship or enmity, personal experience, and unequal probabilities obtained by decisions of other agents in the past. To model the different types of bias behavior, we simulate each node in the BN structure as a complex wave function, including amplitude and phase. The phase parameters model initial biases in the human mind. So the interference term, as a function of initial phase parameters, is estimated by proposing a bias potential function and a new quantum-like entangled witness in Hilbert space. Also, the relation between this witness and Shannon entropy as a measure of entanglement is presented. Empirical results of two decision-making scenarios are used to evaluate our model. One of them is the prisoner's dilemma (PD), a well-known benchmark in this domain with equal probabilities in the first node. And the second one is a categorization and decision-making task based on human faces, with unequal probabilities in the first node. These evaluations confirm the superiority of the proposed model in predicting human selections compared with the classical BN (CBN) and six predictive quantum-like models.

The rest of this paper is organized as follows. Preliminaries about the classical BN, quantum probability, and the previous ideas about merging these two approaches are reviewed. Then we propose a biased entangled QBN approach as a predictive probabilistic decision theory. After that, evaluations of the proposed model on two different scenarios in the literature are presented. Finally, conclusions and recommendations for future works are presented in the last section.

## Preliminaries

### Classical Bayesian Network

Real-world agents usually work together as a system rather than acting individually. Understanding the agent's relations is an unavoidable step for reasoning and decision-making effectively. Bayesian networks are powerful graphical tools for modeling relations between agents using directional arcs between parent and child nodes. This structure provides a compact probabilistic representation of the joint distribution of a set of nodes modeled by random variables (${X}_{i}$). The inference is estimated based on the Bayes' rule:

1 $$\mathit{Pr}(X|Y)=\mathit{Pr}(Y|X)\mathit{Pr}(X)/\mathit{Pr}\left(Y\right).$$

The probability of child nodes are calculated using conditional probability distributions as follows^44^ :

2 $$Pr\left({X}_{1},{X}_{2},\dots ,{X}_{n}\right)=\prod_{i=1}^{n}Pr({X}_{i}\left|parents\left({X}_{i}\right)\right).$$

**Figure 1:**
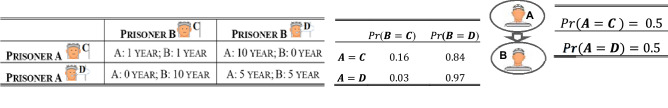
An example of the payoff matrix and classical Bayesian network for the prisoner's dilemma game. Each node is related to a prisoner. The conditional probabilities are presented in related tables in this structure.

Also, probabilities under uncertainty are estimated using the TPL. Let us denote the observed node by ($O$) and unobserved ones by ($U$). The inference for some query $X$ is given by^44^:

3 $$\mathit{Pr}\left(X|O\right)=\alpha Pr(X,O)=\alpha {\sum }_{u\in U}Pr(X,O,u),$$

where $${\alpha }$$ is a normalization factor that guarantees the $$Pr(X|O)$$ adds up to 1. Because of the confluence of artificial intelligence and statistics, Bayesian Networks are becoming increasingly popular in different areas including medicine, engineering, social sciences, economics, and many more^4^. An example of a simple BN structure for a prisoner's dilemma (PD) game, the famous benchmark in the decision-making field, is presented in Fig. 1. In this game, two prisoners are separated in isolation cells. Each prisoner can choose between two options of either cooperating or defecting with another prisoner. Figure 1 presents an example of the payoff matrix for the PD game. Before the second prisoner ($$B)$$, makes a decision, he/she is informed that the first prisoner $$(A)$$ has chosen defection ($$D$$), cooperation ($$C$$), or ($$B)$$ has no information about the first prisoner’s decision (Unknown). Prisoner $$(A)$$ has an equal probability of defecting or cooperating. $$Pr(B=D)$$ is estimated based on the empirical results shown in Table 1 and the TPL rule:

**Table 1:** The probability of choosing defection by a second player according to average results of different versions of PD task reported in^12^.

	Known to		Under uncertainty	
	Defect	Cooperate	Experimental	CP (TPL)
Shafir and Tversky^45^	0.97	0.84	0.63	0.905
Li and Taplini^46^	0.82	0.77	0.72	0.795
Busemeyer et al.^47^	0.91	0.84	0.66	0.875
Hristova and Grinberg^48^	0.97	0.93	0.88	0.950

4 $$\mathit{Pr}\left(B=D\right)=\mathit{Pr}\left(D,D\right)+\mathit{Pr}\left(C,D\right)=\mathit{Pr}\left(B=D|A=D\right)\mathit{Pr}\left(A=D\right)+\mathit{Pr}\left(B=D\right|A=C) \mathit{Pr}\left(A= C\right).$$

As shown in Table 1, the obtained value by (4) does not match the experimental results. TPL has been violated in other experimental observations under uncertainty repeatedly^15^. Thus, despite the impressive capabilities of BNs in inferring and predicting, the violation of TPL challenges the application of this method in predicting human behavior under uncertainty. The similarities between these inconsistencies with some contradictions in classical physics, such as Young's two-slit experiment and the ability of QP to model these discrepancies, have inspired the use of quantum probability in Bayesian networks^12^.

### Introduction to quantum probability

Around the 1930s, two different sets of axioms were formulated for probability theory. One of them is the Kolmogorov axioms, which are the basis of CP^49^. The second one is the Von Neumann axioms, which are the basis of QP^50^. A fundamental difference between classical and quantum probability theory is that CP is represented by the set theory, while QP is founded upon the Hilbert vector space^51^. QP was initially used in quantum computing, but recently the range of its applications has expanded considerably. The equivalent of a classical bit in QIT is called the qubit. The spin of an electron is an example of a qubit. In quantum computing, two eigenstate vectors $$|0\rangle ={\left(\begin{array}{cc}1& 0\end{array}\right)}^{T}$$ and $$|1\rangle ={\left(\begin{array}{cc}0& 1\end{array}\right)}^{T}$$ are defined to model the up and down spin states^52^. Before measurement (under an uncertainty situation), the state of a qubit can be represented as a linear combination of $$|0\rangle$$ and $$|1\rangle$$ in a two-dimensional Hilbert space ($${C}^{2})$$ as follows:

5 $$|\psi \rangle ={c}_{1}{e}^{i{\theta }_{1}}|0\rangle +{c}_{2}{e}^{i{\theta }_{2}}|1\rangle ={\left(\begin{array}{cc}{c}_{1}{e}^{i{\theta }_{1}}& {c}_{2}{e}^{i{\theta }_{2}}\end{array}\right)}^{T}$$

So we deal with the complex coefficients, including amplitude and the phase. A unique feature of QM is that a qubit can be simultaneously in states $|0\rangle$ and $|1\rangle$.

However, when a measurement is done $|\psi \rangle$ will collapse into $|0\rangle$ or $|1\rangle$. ${({c}_{1})}^{2}$ and ${({c}_{2})}^{2}$ are the probability of finding the system in $|0\rangle$ and $|1\rangle$, respectively. $|\psi \rangle$, which is known as the initial state vector, is formed based on our knowledge about the initial state of a system. For each event $|i\rangle$ ($i$=0,1), a projection operator ${P}_{i}=|i\rangle \langle i|$ is assigned to reduce the state vector $|\psi \rangle$ into an eigenstate $|i\rangle$.The probability of finding the system in the state $|i\rangle$ is obtained by Eq. (6)^52^:

6 $$Pr\left(|i\rangle \right)={\left|{P}_{i}|\psi \rangle \right|}^{2}=\langle \psi \left| {P}_{i}\right| \psi \rangle .$$

We can extend these concepts to higher dimensions using the tensor product of $|0\rangle$ or $|1\rangle$ ^52^. For example, the state of a system including two qubits can be represented by a superposition of the following four vector states:

7 $$|00\rangle =|0\rangle \otimes |0\rangle ={(\begin{array}{cc}\begin{array}{cc}1& 0\end{array}& \begin{array}{cc}0& 0\end{array}\end{array})}^{T}; |01\rangle =|0\rangle \otimes |1\rangle ={(\begin{array}{cc}\begin{array}{cc}0& 1\end{array}& \begin{array}{cc}0& 0\end{array}\end{array})}^{T};|10\rangle =|1\rangle \otimes |0\rangle ={(\begin{array}{cc}\begin{array}{cc}0& 0\end{array}& \begin{array}{cc}1& 0\end{array}\end{array})}^{T}; |11\rangle =|1\rangle \otimes |1\rangle ={(\begin{array}{cc}\begin{array}{cc}0& 0\end{array}& \begin{array}{cc}0& 1\end{array}\end{array})}^{T}.$$

8 $$|\psi \rangle ={c}_{1}{e}^{i{\theta }_{1}}|00\rangle +{c}_{2}{e}^{i{\theta }_{2}}|01\rangle +{c}_{3}{e}^{i{\theta }_{3}}|10\rangle +{c}_{4}{e}^{i{\theta }_{4}}|11\rangle ={\left(\begin{array}{cc}{c}_{1}{e}^{i{\theta }_{1}}& \begin{array}{cc}{c}_{2}{e}^{i{\theta }_{2}}& \begin{array}{cc}{c}_{3}{e}^{i{\theta }_{3}}& {c}_{4}{e}^{i{\theta }_{4}}\end{array}\end{array}\end{array}\right)}^{T}.$$

### Quantum-like Bayesian Network

Quantum-like Bayesian networks achieve a significant capacity by merging the ability of QP in dealing with uncertainty and powerful features of Bayesian networks in certain situations. In 1995, Tucci^53^ applied quantum probability in the BN structure for solving a problem in the physical domain. In their approach, each probability function ${Pr}_{n}$ in the real space is replaced by a complex wave function ${\psi }_{n}=\sqrt{{Pr}_{n}}{e}^{i{\theta }_{n}}$ where ${\theta }_{n}$ is a phase factor. But Tucci does not provide a solution for estimating the phase parameters. By considering different values for the phase factor, every CBN can be represented as an unlimited number of QBNs. Here, we present an example of QBN for the PD task. As shown in^11^, the probability of choosing defection by the second prisoner $(B)$ is estimated by applying the quantum counterpart of Eqs. (1–3) and Born rule^52^ in QIT.

9 $$\begin{aligned} \Pr \left( {B = D{ }} \right) & = \alpha \left( {\left| {\psi \left( {D,D} \right) + \psi \left( {D,D} \right)} \right|^{2} } \right) = \alpha \left( {\left| {\psi \left( {D,D} \right){ }} \right|^{2} + \left| {\psi \left( {C,D} \right){ }} \right|^{2} + 2\left| {\psi \left( {D,D} \right){ }} \right|\left| {\psi \left( {C,D} \right){ }} \right|\cos \left( \theta \right)} \right) \\ & = \alpha \left( {Pr\left( {D,D} \right) + Pr\left( {C,D} \right) + interference\;term} \right) \\ \end{aligned}$$

where $\alpha$ is a normalizations factor which is obtained by (10):

10 $$\alpha =\frac{1}{\sqrt{\mathit{Pr} \left(B= D\right)+\mathit{Pr} \left(B= C\right)}}.$$

Similar to considering wave-particle duality in Young's two-slit experiment, the wave behavior is removed in certain situations. However, there may be destructive or constructive interferences under uncertainty if at least two wave functions interact with each other. There were different attempts to describe Young's two-slit experiment by non-Kolmogorovean probabilistic model such as proposing the p-adic theory of probability^54^. Inspired by this experiment, Khrennikov^55^ introduces the measure of statistical perturbations and considers interference terms in total probability law. He proposes the Quantum-like Representation Algorithm (QLRA) and modifies the classical TPL by assuming a trigonometric or hyperbolic interference term^56^. Considering the interference effects in TPL is a key idea to justify the contradictions in most quantum-like decision models. Including this effect is also the most important challenge of these models to provide predictions of human selections.

Most quantum-like decision theories only justify the observations by selecting suitable interference-effect. While implementing the predictive decision theory needs to estimate the interference term instead. Quantum decision theory (QDT)^57^ is one of the first predictive approaches for estimating the interference term. This model takes into account objective utility related to expected benefit, as well as subjective interference. By applying some quantum parameters, this model assumes the interference term at a static value of 0.25. Wang et al.^25^ show that Bayesian and Markov models cannot account for empirical data on human behavior. In addition to justifying empirical data of human selections by considering a static value of interference term for each scenario, they present a predictive QQ model which is supported by empirical tests of order effects. After that, Moreira et al.^14^ added a predictive feature to the QBN structure using a piecewise heuristic function based on probabilities in the equivalent CBN. In 2019, Huang et al.^58^ presented a solution based on the belief entropy concept. They use Deng entropy^59^ to estimate the interference term, which is a generalized form of Shannon entropy and is defined as^59^:

11 $${E}_{d}\left(m\right)=-\sum_{A\subseteq X}m\left(A\right){\text{log}}_{2}\left(m\left(x\right)/{(2}^{\left|A\right|}-1)\right).$$

where $m$ is a mass function defined on $X$, $A$ is the focal element of m and $\left|A\right|$ is equal to the cardinality of $A$ ^59^. Their model has some drawbacks leading to an estimated value of more than one for the cosine function in the interference term^27^. So, according to probability values in certain situations presented in BN tables, it is possible to achieve the unadmitted complex number for probability in some cases^27^. Another heuristics approach is proposed by Dia et al. ^60^. In this method, a coefficient $r$, based on wave functions in QBN, is defined to estimate the interference term. At the same time, Wichert et al.^61^ propose the Balanced Quantum-Like Bayesian Networks. They introduce the law of balance based on the notion of balanced intensity waves and the law of maximum uncertainty based on the entropy concept. The main idea of this method is to balance the intensity waves resulting from quantum interference in such a way that, during Bayes normalization, they cancel each other. Finally, a predictive entangled QBN (PEQBN) structure is introduced in our previous study^27^, motivated by the QIT instead of heuristics approaches. Entangled nodes in the PEQBN model mean that each node/DM is considered a part of the whole system. Therefore, the effect of indirect relations that cannot be modeled as classical arcs are estimated by two entanglement measures in QIT, and the proposed $qW$ witness^27^.

## Method

Here we present a biased variant of entangled quantum-like BN from a cognitive perspective to examine bias behavior in the decision-making process. We consider different bias behaviors due to the emotions between agents or past personal experiences motivated by the entanglement concept. Besides, the effect of unequal probabilities in the parent node, obtained from past experiences of other agents, is modeled that is inspired by electric fields. We simulate a two-step binary decision-making experiment in an entangled quantum-like BN structure^27^ and predict the probabilities in the child nodes without any observations of parent nodes. The dynamical evolution of selecting each choice under uncertainty is simulated by overlapped wave functions^12^ that cause an interference effect because of the existence of phase parameters.

The problem of justifying the phase parameter from the perspective of social systems is the most important unsolved challenge of quantum-like models. Providing an acceptable justification for these parameters can pave the way to estimating the interference terms and proposing predictive quantum-like models. The phase parameters in the superposition state of a quantum system are related to the environmental condition and obtained from the initial experimental setting in a physical system. Although these parameters are unknown in social systems, they are attributed to the contextual conditions of the DM^33^, the correlation between the phenomena^42^, or the degree of uncertainty of the DM^62^ to the decision scenario, in different quantum-like models. In this study, we attribute the phases in the superposition state to the initial mental conditions of the decision-maker, which is a function of personal experiences, spiritual conditions, and the decision-maker's relations with the other agents in the society. So we use these parameters to mode bias behavior in the human mind and unknown relationships between agents. In this regard, the introduced social entanglement concept, inspired by quantum entanglement in our previous study^27^, has a key role in our method. This means that although we are not dealing with a quantum system, the concept of quantum-like entanglement has been borrowed from quantum physics due to the similarity between entangled quantum systems and social systems. Similar to entangled quantum systems, information about the social system is not complete and the information is shared in the whole system. Also, as it is not possible to separate the information of each particle in an entangled quantum system, decision-makers in a social system are not considered isolated agents. Unknown relations between agents influence the decision-making process, and so it is impossible to measure the initial mental conditions of each decision-maker separately.

On the other hand, as quantum interference is related to entanglement in quantum physics, we assume that the interference term in the social system is related to the social entanglement. The fundamental differences between quantum and classical interference arise from non-separability in quantum systems which leads to interference between probability amplitudes rather than between physically existing realistic objects, like the electromagnetic waves ^57,63^. There are several common properties between decision-making processes and quantum interference. The intention interference and non-commutativity of subsequent decisions are critical characteristics of human decision-making. The non-commutativity of actions (in the framework of quantum operators) and entanglement are essential properties of quantum interference. In contrast, classical interference cannot include these effects in the system due to its commutative nature and locality. So, as assumed in our previous PEQBN model, we relate the interference term to the entanglement between agents^27^ $E\left(\rho \right):$ 

12 $$cos \left(\theta \right)=-E\left(\rho \right).$$

where $\rho =\left|\psi > <\psi \right|$. In QIT, entanglement considers long-range effects because of uncertainty sources in the dynamical behavior of physical system. Although there is no exact tool for measuring the entanglement of a quantum composite system, there are many criteria, known as quantum measures, to estimate entanglement values or at least find the boundaries of this parameter. In addition, there are necessary and sufficient entanglement criteria in terms of directly measurable observables, called entanglement witnesses^64^. Because entanglement witnesses are directly measurable quantities, they are very useful for the experimental analysis of entanglement^64^. So, because the initial conditions of social systems are not well known, we cannot estimate entanglement measures directly, and we introduce a measurable function as a quantum-like witness of social entanglement. Then we obtain its relations to the concurrence entanglement measure, based on empirical data, to estimate entanglement in the social system and hence the interference term.

### Modeling the bias behavior inspired by the entanglement concept

This section introduces a new quantum-like entangled witness in Hilbert space for modeling bias behavior caused by emotions such as friendship or enmity between agents or past personal experiences from a cognitive viewpoint. It is noted that bias is usually interpreted as prior information in Bayes' formula. Still, this interpretation only considers the effect of bias behavior on the amplitude of wave function in a QBN structure. In this study, we consider the effect of bias behavior on phase parameters too. So, we assume that prior information such as past experiences or relationships with other agents can change the decision-maker's initial state, including amplitude and phase parameters.

**Figure 2:**
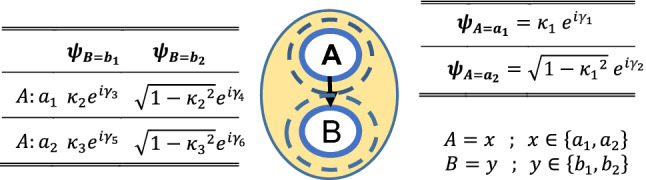
An entangled bayesian network including two nodes. Each node (A and B) is considered a part of the whole system (illustrated by a yellow oval). The nodes in the social system are entangled due to unknown sources such as trust and cooperation between agents. In the BEQBN, probabilities in BNs are replaced by suitable complex wave functions.

Here we explain the proposed model by considering a BN including two nodes $$(A$$ and $$B$$) in Fig. 2. These nodes are entangled as well as related to each other by a classical arc. The wave functions presented in Fig. 2 are obtained based on empirical probability values in the absence of uncertainty. In QIT, each quantum event in QP is modeled by a state vector in Hilbert space. In a binary choice, two options for each node, including $$|{a}_{1}\rangle ={\left(\begin{array}{cc}1& 0\end{array}\right)}^{T}$$ and $$|{a}_{2}\rangle ={\left(\begin{array}{cc}0& 1\end{array}\right)}^{T}$$, are defined in Hilbert space $${H}_{1}$$. For a two-stage problem, we also can define $$|{b}_{1}\rangle ={\left(\begin{array}{cc}1& 0\end{array}\right)}^{T}$$ and $$|{b}_{2}\rangle ={\left(\begin{array}{cc}0& 1\end{array}\right)}^{T}$$ in Hilbert space $${H}_{2}$$. So for extending the decision-making problem to the four dimensions, the composite Hilbert space ($$H)$$ is formed based on four basis eigenvectors obtained by the tensor product^52^:

13 $$H = H_{1} \otimes H_{2} { };\quad \left| {a_{i} b_{j} } \right\rangle = \left| {a_{i} } \right\rangle \otimes \left| {b_{j} } \right\rangle .$$

The above Hilbert space represents all possible results after a measurement. So until we are not informed about the results of a decision-making process (doing a measurement) in nodes $A$ and $B$, the superposition state is formed as follows:

14 $$|\psi \rangle ={c}_{1}{e}^{i{\theta }_{1}}|{a}_{1}{b}_{1}\rangle +{c}_{2}{e}^{i{\theta }_{2}}|{a}_{1}{b}_{2}\rangle +{c}_{3}{e}^{i{\theta }_{3}}|{a}_{2}{b}_{1}\rangle +{c}_{4}{e}^{i{\theta }_{4}}|{a}_{2}{b}_{2}\rangle ={\left(\begin{array}{cc}\begin{array}{cc}{c}_{1}{e}^{i{\theta }_{1}}& {c}_{2}{e}^{i{\theta }_{2}}\end{array}& \begin{array}{cc}{c}_{3}{e}^{i{\theta }_{3}}& {c}_{4}{e}^{i{\theta }_{4}}\end{array}\end{array}\right)}^{T}$$

where ${c}_{i}$ and ${\theta }_{i}$ are defined in Table 2. The first step for estimating the $Pr(B={b}_{1})$ without measuring $A$ is applying the projector ${P}_{B={b}_{1}}=|{b}_{1}\rangle \langle {b}_{1}|$ on the superposition state $|\psi \rangle$.

15 $$\begin{aligned} Pr \left( {B = b_{1} } \right) & = \left| {P_{{B = b_{1} }} |\psi\rangle } \right|^{2} = \langle\psi \left| {{ }P_{{B = b_{1} }} } \right|\psi\rangle = \left( {|b_{1}\rangle \langle b_{1} {|}\psi\rangle } \right) ^\dagger \left( {|b_{1} \rangle \langle b_{1} {|}\psi\rangle } \right) \\ & = \left( {\left| {\langle b_{1} {|}a_{1} \rangle \langle a_{1} {|}\psi \rangle} \right|^{2} + \left| {\langle b_{1} {|}a_{2}\rangle \langle a_{2} {|}\psi \rangle} \right|^{2} + \left( {\left| {\langle b_{1} {|}a_{1}\rangle } \right|\left| {\langle a_{1} {|}\psi\rangle } \right|\left| {\langle \psi {|}a_{2}\rangle } \right|\left| {\langle a_{2} {|}b_{1}\rangle } \right|cos \hspace{0.2em} \theta } \right)} \right) \\ & = c_{1}^{2} + c_{3}^{2} + \left( {\left| {\langle b_{1} {|}a_{1} \rangle} \right|\left| { \langle a_{1} {|}\psi\rangle } \right|\left| {\langle\psi {|}a_{2}\rangle } \right|\left| {\langle a_{2} {|}b_{1} \rangle} \right|cos \hspace{0.2em} \theta } \right). \\ \end{aligned}$$

**Table 2:** Wave functions in the BEQBN model presented in Fig. 2.

$A$	$B$	$\psi (A, B)$
${a}_{1}$	${b}_{1}$	$\psi \left({a}_{1},{b}_{1}\right)={\kappa }_{1} {e}^{i{\gamma }_{1}} {\kappa }_{2} {e}^{i{\gamma }_{3}}={c}_{1}{e}^{i{\theta }_{1}}$
${a}_{1}$	${b}_{2}$	$\psi \left({a}_{1},{b}_{2}\right)={\kappa }_{1} {e}^{i{\gamma }_{1}} \sqrt{1-{{\kappa }_{2}}^{2}}{ e}^{i{\gamma }_{4}}={c}_{2}{e}^{i{\theta }_{2}}$
${a}_{2}$	${b}_{1}$	$\psi \left({a}_{2},{b}_{1}\right)=\sqrt{1-{{\kappa }_{1}}^{2}} {e}^{i{\gamma }_{2}}{\kappa }_{3} {e}^{i{\gamma }_{5}}={c}_{3}{e}^{i{\theta }_{3}}$
${a}_{2}$	${b}_{2}$	$\psi \left({a}_{2},{b}_{2}\right)=\sqrt{1-{{\kappa }_{1}}^{2}} {e}^{i{\gamma }_{2}}\sqrt{1-{{\kappa }_{3}}^{2}} {e}^{i{\gamma }_{6}}={c}_{4}{e}^{i{\theta }_{4}}$

This equation is obtained by applying Eq. (6) on the wave function $|\psi \rangle$. The third term in Eq. (15) is related to the interference effect, which adds a stochastic long-range impact to the problem. If we remove the uncertainty from the first node by doing an observation, the third term becomes equal to zero. So, $\text{Pr}\left(B={b}_{1}\right)$ becomes equal to ${{c}_{1}}^{2}$ or ${{c}_{3}}^{2}$ according to the result of measuring $A$. Now let us consider the unknown situations in which there is no information about node $A$. In this case, we need to estimate the interference term related to the initial condition in the superposition state/initial bias in mind. The key parameter of the interference term is $cos (\theta )$, which is a function of initial phase parameters, used for modeling bias behavior in this study. So, inspired by physical systems, we relate the interference term to the initial experimental situation. But in social system, initial experimental situations are the function of experiences of DM or unknown relations between agents in the society. Inspired by the entanglement concept in quantum physics, which models the unknown relation between particles in a whole system, we introduce a similar concept called *social entanglement* in a quantum-like multi-agent system that is only partially known. So, here we simulate the social system as a composite quantum system. Then we attribute the interference effects to Shannon entropy as a measure of entanglement. Based on quantum studies Shannon entropy (${E}_{Sh}$) is related to the concurrence $(C)$, a common measure of quantum entanglement, as shown below:

16 $${E\left(\rho \right)=E}_{Sh}(\rho )=- m{\mathit{log}}_{2}m-(1-m){\mathit{log}}_{2}(1-m),m=(1+\sqrt{1-{C\left(\rho \right)}^{2}})/2.$$

Because the initial conditions of a social system and density matrix ($\rho$) are not well known, we cannot estimate $E(\rho )$ directly, so an observable function called quantum-like entanglement witness $(qlw)$ is introduced in situations with certainty. By finding the relation between $\left(qlw\right)$ and $(C)$ based on empirical data, we estimate social entanglement in the uncertain case. Afterward, Shannon entropy and the interference term (cos (θ)) are estimated by measuring the system entanglement in a practical way.

Let us review this approach in more details. For estimating this parameter that is inspired by the entanglement concept, a novel quantum-like witness in Hilbert space is introduced in this study. Unlike previous methods, we use a quantum solution in a four-dimensional Hilbert space based on QP for solving a quantum-like problem. In the QBN structure, such as shown in Fig. 2, regardless of the condition in the first node, the uncertainty in the second node is eliminated by observing node $B$.

According to QIT, the operator $I\otimes {P}_{B={b}_{1}}$ tells us to leave the first node unobserved and apply the ${P}_{B={b}_{1}}$ operator to the second node ^52^. Here we define two input vectors ${v}_{1}$, and ${v}_{2}$ as follows:

17 $$\begin{aligned} & v_{1} = I \otimes P_{{B = b_{1} }} | {\psi\rangle = \left( {\begin{array}{*{20}c} {P_{{B = b_{1} }} } & 0 \\ 0 & {P_{{B = b_{1} }} } \\ \end{array} } \right)} |\psi \rangle = \left( {\begin{array}{*{20}l} 1 \hfill & 0 \hfill & 0 \hfill & 0 \hfill \\ 0 \hfill & 0 \hfill & 0 \hfill & 0 \hfill \\ 0 \hfill & 0 \hfill & 1 \hfill & 0 \hfill \\ 0 \hfill & 0 \hfill & 0 \hfill & 0 \hfill \\ \end{array} } \right)\left( {\begin{array}{*{20}l} {c_{1} e^{{i\theta_{1} }} } \hfill \\ {c_{2} e^{{i\theta_{2} }} } \hfill \\ {c_{3} e^{{i\theta_{3} }} } \hfill \\ {c_{4} e^{{i\theta_{4} }} } \hfill \\ \end{array} } \right) = \left( {\begin{array}{*{20}l} {c_{1} e^{{i\theta_{1} }} } \hfill \\ 0 \hfill \\ {c_{3} e^{{i\theta_{3} }} } \hfill \\ 0 \hfill \\ \end{array} } \right), \\ & v_{2} = I \otimes P_{{B = b_{2} }} | {\psi\rangle = \left( {\begin{array}{*{20}c} {P_{{B = b_{2} }} } & 0 \\ 0 & {P_{{B = b_{2} }} } \\ \end{array} } \right)} |\psi \rangle = \left( {\begin{array}{*{20}l} 0 \hfill & 0 \hfill & 0 \hfill & 0 \hfill \\ 0 \hfill & 1 \hfill & 0 \hfill & 0 \hfill \\ 0 \hfill & 0 \hfill & 0 \hfill & 0 \hfill \\ 0 \hfill & 0 \hfill & 0 \hfill & 1 \hfill \\ \end{array} } \right)\left( {\begin{array}{*{20}l} {c_{1} e^{{i\theta_{1} }} } \hfill \\ {c_{2} e^{{i\theta_{2} }} } \hfill \\ {c_{3} e^{{i\theta_{3} }} } \hfill \\ {c_{4} e^{{i\theta_{4} }} } \hfill \\ \end{array} } \right) = \left( {\begin{array}{*{20}l} 0 \hfill \\ {c_{2} e^{{i\theta_{2} }} } \hfill \\ 0 \hfill \\ {c_{4} e^{{i\theta_{4} }} } \hfill \\ \end{array} } \right). \\ \end{aligned}$$

These input vectors are defined in four-dimensional Hilbert space by applying a quantum view to the problem instead of the classical view used to define two-dimensional vectors in other QBN models. Finally, an observable function called quantum-like witness ($qlw$) is suggested based on these vectors as shown below:

18 $$qlw\left({v}_{1},{v}_{2}\right)={\text{cos}}^{-1}\left(\frac{{\left(\left|{v}_{1}-{v}_{2}\right|\right)}^{2}+{\left|{v}_{1}\right|}^{2}-{\left|{v}_{2}\right|}^{2}}{2*\left|{v}_{1}-{v}_{2}\right|*\left|{v}_{1}\right|}\right).$$

For estimating the interference term, a relationship between the introduced $qlw$ witness and $C$ is obtained by applying the S_R_TLBO optimization algorithm^65^. This relationship is defined in a way that optimizes the concurrence as much as possible to achieve the best fit between predicted and experimental probabilities. Applying optimization in this procedure is meaningful because optimization is used in the definition of many quantum entanglement measures and witnesses due to some reasons such as considering the best initial conditions^66^. So we use the following equation to estimate the concurrence entanglement measure:

19 $$C =\sqrt{0.136-0.03\;cos\left(n\; qlw\right)+0.02\; * \mathit{sin}\left(n\; qlw\right)-0.029\; *cos\left(2n\; qlw\right)-0.12\;  *sin\left(2n\; qlw\right).}$$

where $n$ is equal to ${2}^{4}$. This equation guarantees that the $C$ value is obtained between 0 and 1, which is compatible with the acceptable range for this entanglement measure. By applying Eq. (16), Shannon entropy, as a measure of entanglement, is estimated. After that, $cos (\theta )$ in the interference term is obtained by Eq. (12), inspired by QIT.

### Modeling the bias behavior inspired by electric fields

**Figure 3:**
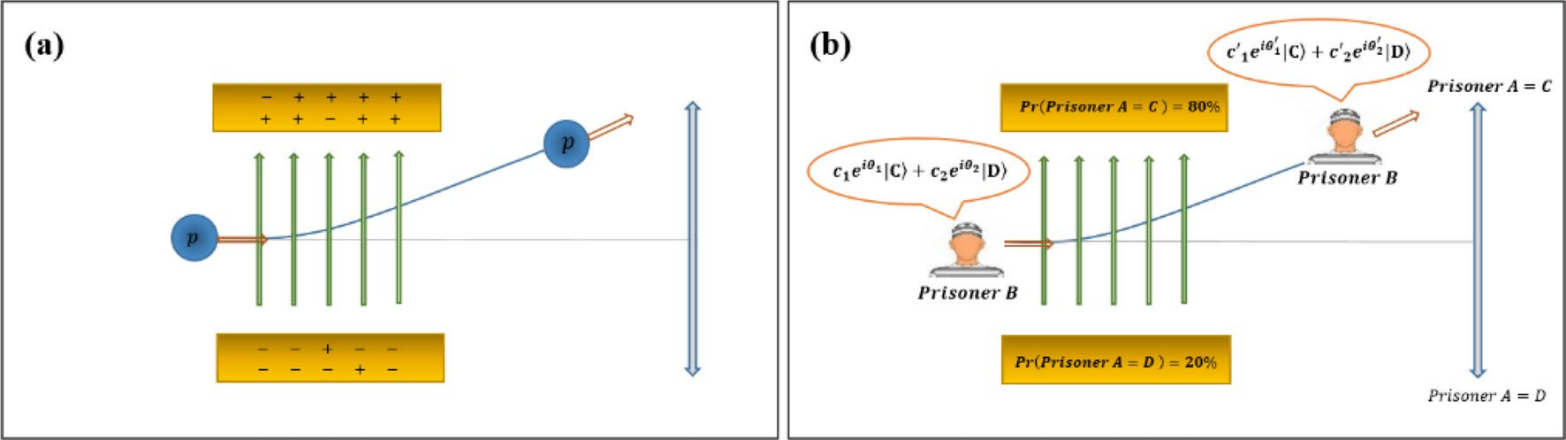
(**a**) The deviation of the proton $p$ under the influence of the electric field, created due to the unequal percentage of positive charges in the plates under the classical view, (**b**) The deviation of the initial superposition state including phase parameters in the human mind of the second prisoner (under unequal probabilities of defection and cooperation of the first prisoner) due to considering a hypothetical bias field under the quantum-like view.

The main advantage of this study is modeling the mind's initial bias in the decision-making process due to unequal probabilities in the parent node obtained from past decisions of other agents in society. In most studies on QBN structures, the quantum-like approaches are evaluated on simple two-step binary decision-making tasks, with equal probability in the parent node, such as prisoner's dilemma and two-step gambling games^67^. Let us focus on situations in which $Pr(a1)\sim =Pr(a2$) in the first node. When a decision between two choices has not been made yet, we think about both options simultaneously, and a superposition state is formed in the QBN structure. The coefficients of each state in the superposition are complex numbers. Unequal probabilities affect the amplitude of these coefficients, but we also consider the effect of unequal probabilities on the initial phase. For modeling this effect, we are inspired by the deviation of the proton during the passage between two plates. Imagine some electrons being transferred from one plate to another. In this case, the percentage of positive charges of each plate changes from 50%, and an electric field is created between two charged plates, which diverts the passing proton to one of the plates (Fig. 3a). Similarly, imagine that the probability of one option in a binary problem is more than 50%, despite being unaware of the results, the initial condition of our mind tends toward the option with a higher probability. In this regard, we propose a hypothetical bias potential field to model this bias behavior on initial phase parameters (Fig. 3b).

**Figure 4:**
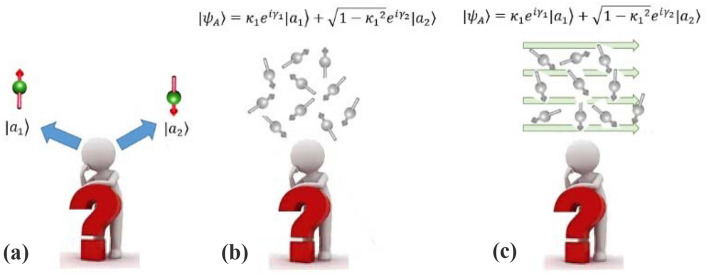
According to BEQBN, humans made their decisions by interpreting the information for each choice separately in the absence of uncertainty (**a**) or by thinking about two choices simultaneously (**b** and **c**) under uncertainty. Also, this model considers a biased decision-making process inspired by electric fields when the probabilities of finding the system in two options are not equal (**c**).

**Figure 5:**
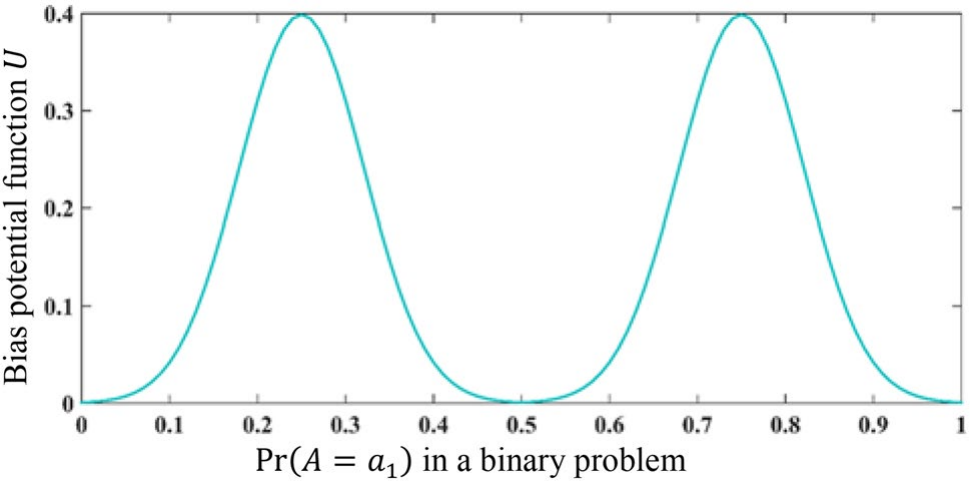
The proposed bias potential function Eq. (21) for modeling biased decision-making. The value of this function is added to the interference term. *U*(0.5) ≈ 0, due to an unbiased situation in the case of finding equal probabilities in the first node. Also, *U*(0) = *U*(1) ≈ 0 because the interference term is removed in certain situations.

Classical models consider that DMs think about each option separately (Fig. 4a), even in the presence of uncertainty in node A (parent node). The proposed method considers this situation, only if uncertainty is removed by an observation. While from a quantum-like viewpoint, node $A$ can exist in the following superposition state (Fig. 4b) under uncertainty:

20 $$|{\psi }_{A}\rangle ={\kappa }_{1}{e}^{i{\gamma }_{1}}|{a}_{1}\rangle +{\kappa }_{2}{e}^{i{\gamma }_{2}}|{a}_{2}\rangle ={ \left(\begin{array}{cc}{\kappa }_{1}{e}^{i{\gamma }_{1}}& {\kappa }_{2}{e}^{i{\gamma }_{2}}\end{array}\right)}^{T}.$$

where ${\kappa }_{2}=\sqrt{1-{{\kappa }_{1}}^{2}}$. The effect of unequal probabilities has appeared in the amplitude parameters ${(\kappa }_{\text{i}})$. However, phase parameters ${(\gamma }_{\text{i}})$ and, therefore, interference terms are also affected, indirectly. When a DM wants to choose an option in the second node that can be beneficial or detrimental, both options with unequal probabilities in the first node are considered simultaneously (Fig. 4b). Here, we suggest that DM's mind tends to the event that is more likely to happen. Since we attribute the initial phases to the DM's mental conditions, we need to model the effect of this bias on ${(\gamma }_{\text{i}})$ parameters and, therefore, the interference term. We model the bias effect to initial phases by a hypothetical field motivated by the behavior of particles in an electric field (Fig. 4c). So most DMs/qubits tend to have a particular bias. In this regard, a potential function $U({\kappa }_{1})$ is defined to model bias behavior (Fig. 5), and the supplementary term shown in Eq. (21) is added to Eq. (15):

21 $$U\left({\kappa }_{1}=\sqrt{Pr\left(A={a}_{1}\right)}\right)=\left\{\begin{array}{c}\vspace{0.3cm}\displaystyle{\frac{{e}^{-100 \,({ {\kappa }_{1}}^{2}-0.75)^{2}}}{\sqrt{2\pi }} }\qquad Pr(A={a}_{1})\ge 0.5 \\ \displaystyle{\frac{{e}^{-100 \,({ {\kappa }_{1}}^{2}-0.25)^{2}}}{\sqrt{2\pi }} }\qquad Pr(A={a}_{1})<0.5\end{array}\right.\;.$$

If two options have equal probabilities, no significant bias is observed and $U\left(\sqrt{0.5}\right)\approx 0$. Also, $U(0)= U(1)\approx 0$ because there is no uncertainty in these situations. But if the probability of choosing one option is 0.75 and the other one is 0.25, $U({\kappa }_{1})$ is maximized. The proposed bias function (Fig. 5) is symmetric because the probability of one option $Pr(A={a}_{1})$ is equivalent to the probability value of $(1- Pr(A={a}_{1}) )$ for the second option. So, we can predict $Pr(B={b}_{1})$ without measuring $A$ as follows:

22 $$\mathit{Pr}\left(B={b}_{1}\right)={\left|{P}_{B={b}_{1}}|\psi\rangle \right|}^{2}=\min\{1,{{c}_{1}}^{2}+{{c}_{3}}^{2}-\left(\left|\langle {b}_{1}|{a}_{1}\rangle \right|\left|\langle {a}_{1}|\psi \rangle \right|\left|\langle \psi |{a}_{2}\rangle \right|\left|\langle {a}_{2}|{b}_{1}\rangle \right|{*\; E}_{Sh}\right)+U\left({\kappa }_{1}\right)\}\,.$$

The pseudo-code of the proposed BEQBN method is presented in Table 3.

**Table 3:** The Pseudo-code of the BEQBN model for a binary decision-making problem.

Step 1: Two vectors for states of each binary node are presented as $$|{a}_{1}\rangle ={\left(\begin{array}{cc}1& 0\end{array}\right)}^{T}$$ and $$|{a}_{2}\rangle ={\left(\begin{array}{cc}0& 1\end{array}\right)}^{T}$$ in Hilbert space ${H}_{1}$ and $$|{b}_{1}\rangle ={\left(\begin{array}{cc}1& 0\end{array}\right)}^{T}$$ and $$|{b}_{2}\rangle ={\left(\begin{array}{cc}0& 1\end{array}\right)}^{T}$$ in ${H}_{2}$
Step 2: The composite Hilbert space $H = H_{1} \otimes H_{2}$ and four basis vectors are obtained by applying the tensor product
Step 3: Superposition model $|\psi \rangle$ is formed as presented in Eq. (14)
Step 4: Two input vectors ${v}_{1}$, and ${v}_{2}$, are obtained by Eq. (17)
Step 5: Quantum-like witness ($qlw$) is calculated by Eq. (18)
Step 6: The concurrence entanglement measure ($C$ ) is calculated by Eq. (19)
Step 7: Shannon entropy ${E}_{Sh}$ is calculated by Eq. (16)
Step 8: The biased operator $U$ is estimated by Eq. (21)
Step 9: The projector ${P}_{B=1}$ is applied on $|\psi \rangle$, and $\text{cos}\left(\theta \right)$ in Eq. (15) is considered equal to $-{E}_{Sh}.$
Step 10: ${Pr}(B={b}_{1})$ is estimated by Eq. (22)

## Results

Here, the proposed BEQBN is evaluated on two decision-making scenarios with equal and unequal probabilities in the first node. To provide a fair comparison, Obtained results by BEQBN compare to the estimated value by CBN and six quantum-like decision-making models.

### Evaluation of the proposed BEQBN on PD game

**Figure 6:**
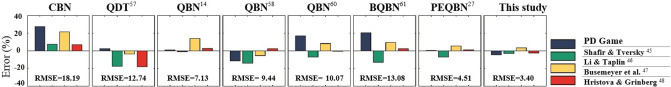
Graphical illustration of errors between empirical results (Table 1) and predicted values of $\text{Pr}\left(\text{B}=\text{D}\right)$. The results are obtained by applying seven models, including CBN, six recent quantum-like decision models, and the proposed BEQBN model in this study to the PD game.

The PD game is a benchmark in the decision-making domain with equal probabilities in the first node, described in more detail in the preliminaries section. We apply the proposed model, CBN, and six other predictive quantum-like methods, including QDT^57^, QBN^14^, QBN^58^, QBN^60^, BQBN^61^, and PEQBN^27^, to different versions of the PD game. This evaluation is based on the empirical results reported in Table 1. The predicted probability for choosing the defect option by the second player is estimated by each method. The errors between predicted and empirical probabilities for these approaches are reported in Table 4. Also, these results along with their root mean square error ($\text{RMSE}$) values are plotted in Fig. 6. According to this evaluation, the predictions by the proposed BEQBN model are in good agreement with the empirical data. Due to considering bias behavior, BEQBN achieves the first rank in this evaluation by obtaining $\text{an RMSE}$ value equal to 3.4.

**Table 4:** Errors between predicted probability and empirical results for $\text{Pr}\left(\text{B}=\text{defect}\right)$ in the PD game under uncertainty. Predicted values are obtained by applying the BEQBN model CBN, QDT^57^, QBN^14^, QBN^58^, QBN^60^, BQBN^61^, and QBN^27^ to the PD game.

PD game	CBN	QDT^57^	QBN^14^	QBN^58^	QBN^60^	BQBN^61^	PEQBN^27^	The proposed BEQBN
Shafir and Tversky^45^	27.50	2.50	1.08	− 11.16	17.01	20.57	**0.54**	− 4.54
Li and Taplin^46^	7.50	− 17.50	**− 0.78**	− 14.07	− 6.96	− 12.84	− 6.98	− 2.98
Busemeyer et al.^47^	21.50	− 3.50	13.95	− 5.31	8.25	9.5	5.58	**3.40**
Hristova and Grinberg^48^	7.00	− 18.00	2.64	2.45	**− 0.51**	2.42	1.14	− 2.33
$\text{RMSE}$	18.19	12.74	7.13	9.44	10.07	13.08	4.51	**3.40**

Significant values are in [bold].

Because of the equal probability of defection or cooperation of the first prisoner, mental bias modeled by bias potential function is neglected in this task. However, mental bias due to emotions between two prisoners is modeled by the $qlw$ witness and two entanglement measures. This effect is justified by the previous knowledge of the two prisoners of moral character and cooperation between them, although they have been in separate cells. Our previous entangled BN^27^ and the heuristic method presented by Moreira et al.^14^, rank second to third in this evaluation, respectively. Other compared approaches, including QBN^58^, QBN^60^, QDT^57^, BQBN^61^, and classical BN, are ranked fourth to eighth.

### Evaluation of BEQBN on face categorization and decision

In the second evaluation, the proposed model is applied to a two-step scenario with unequal probabilities in the first node, while selection in the second node has benefit or loss for the DM. This task is presented by Busemeyer et al.^68^ about categorizing people based on their faces. In this task, there are two separated conditions: (1) categorization before decision-making (C-D), and (2) decision-making (D alone).

Same pictures of faces are shown to participants in both situations that varied in two dimensions: face width and lip thickness. Participants in the C-D condition are asked to classify the face as either a "good" or "bad" and then choose whether to "attack" or "withdraw." Faces are classified into two groups: "narrow faces" with a narrow breadth and thick lips and "wide faces" with a wide breadth and thin lips. The participants are informed that "narrow" faces have a 0.83 chance of being in the "bad guy" population, whereas "wide" faces have a 0.84 probability of being in the "good guy" population. Attacking the "bad man" and retreating from the "good guy" are rewarded. The participants in the D alone condition are instructed to decide without any classifications.

We apply the proposed BEQBN and seven other predictive methods, including CBN, QDT^57^, QBN^14^, QBN^58^, QBN^60^, BQBN^61^, and PEQBN^27^, to this scenario and predict the probability of choosing "attack" in the D alone condition based on information presented in Table 5. According to our approach, categorization in the first round changes the initial conditions of the DM's mind and therefore the superposition state of each DM. So categorization and decision-making in this task are two entangled processes. In D alone scenario, participants think about categorization as good or bad guys simultaneously. The interference term is estimated by entanglement measures and caused by changing the initial state of the mind in the categorization process. Another human bias is also considered, due to the unequal probabilities of whether each face is good or bad in the first node. Decision-makers think simultaneously about two options, but they are more inclined towards one of them. This bias is modeled by adding the potential function $U$ introduced in Eq. (21) As shown in Table 6, this correction reduces the error between predicted and empirical results.

**Table 5:** The probability of taking defensive action under two different situations of the C-D condition and the D-alone condition^12,68^.

	Pr(good)	Categorization then decision-making (C-D)		Decision-making only (D alone)
		(Attack|good)	(Attack|bad)	Experimental (attack)
Wide face	0.84	0.35	0.52	0.39
Narrow face	0.17	0.41	0.63	0.69

**Table 6:** Errors between the empirical results and predicted probability of taking defensive action without categorization based on eight predictive models including CBN, QDT^57^, QBN^14^, QBN^58^, QBN^60^, BQBN^61^, QBN^27^, and the proposed BEQBN model in this study.

	CBN	QDT^57^	QBN^14^	QBN^58^	QBN^60^	BQBN^61^	PEQBN^27^	The proposed BEQBN
Wide face	**− 1.28**	− 26.28	− 15.80	4.24	− 14.96	29.72	− 6.01	7.05
Narrow face	− 9.74	− 34.74	9.89	− 16.85	7.96	− 33.90	− 20.69	**− 1.30**
$\text{RMSE}$	6.94	30.80	13.18	12.29	11.98	31.88	15.24	**5.07**

Significant values are in [bold].

**Figure 7:**
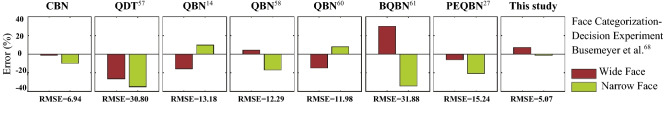
Graphical illustration of errors between empirical results (Table 5) and predicted probability of taking defensive action without categorization. Predicted results are obtained by applying CBN, six quantum-like decision models, and the proposed BEQBN model in this study on Categorization-Decision Experiment^68^.

In this table, BEQBN wins the first rank by achieving the $\text{RMSE}$ value equal to 5.07. The results obtained by other compared methods are shown in Table 6 and Fig. 7. According to these results, the other six quantum-like models are no more successful than classical Bayesian network structure, and CBN, QBN^60^, QBN^58^, QBN^14^, PEQBN^27^, QDT^57^, and BQBN^61^ are ranked second to eighth, respectively. Therefore, in the situations where we deal with unequal probabilities in the first node, considering only one aspect of interference does not improve the performance of BN structures.

## Conclusion

Future autonomous systems will need cognitive capabilities, including reasoning and decision making. Hence, modeling human selection behavior is one of the grand challenges in the future of control and systems. This work extends quantum-like approaches to modeling human choice behavior by focusing on human biases from a cognitive viewpoint. The proposed model is built upon the basis of the entangled quantum-like BN due to the synergy of three powerful approaches, including (1) classical BN for modeling causal relationships, (2) quantum probability for adding a new dimension for modeling the states of the human mind in the complex domain, and (3) entanglement property for modeling the long-range effects due to uncertainty sources in the dynamical behavior of the decision-making process. By simulating a two-step binary choice task as a composite entangled quantum system, we consider each node of a BN structure as a particle or wave in certain and uncertain situations, respectively.

The point of departure in this study is modeling the initial bias behavior of the DM's mind due to unequal probabilities in the unmeasured parent node of the BN structure. To this end, we define a potential function inspired by an electric field to estimate the effect of bias behaviors on the interference effect under uncertainty. Also, a novel quantum-like witness in the Hilbert space is introduced to model bias behavior due to emotions between agents in the social systems and past personal experiences. By applying the proposed quantum-like witness, a quantum solution in Hilbert space rather than classical ones in other models is used to solve a quantum-like problem. We also present a periodic relationship between the proposed quantum-like witness and the well-known concurrence entanglement measure in QIT. Then Shannon entropy is estimated based on the relations in quantum information theory and applied for modeling the destructive or constructive interference effects between the two choices in a binary task under uncertainty.

Finally, the proposed BEQBN is evaluated successfully on two decision-making scenarios with equal and unequal probabilities in the first node. According to experimental results, BEQBN predicts the probability of selecting each option under uncertainty with minimum error compared to classical BN and six recent quantum-like Bayesian networks. The results indicate that the proposed model in this study archives the first rank in overall evaluations by considering mind’s biased behaviors.

In the future, we hope to extend our approach for modeling more complex situations with three different options for each step. For this purpose, we also plan to design new tasks for gathering empirical data and creating new databases. Then, we can evaluate the application of the proposed model in solving human challenges such as crisis management, energy management, or traffic forecasting.

## Data Availability

All data generated or analyzed during this study are included in this published article [and its supplementary information files].
